# Comparative Analysis of Nutrients, Phytochemicals, and Minerals in Colored Sweet Potato (*Ipomoea batatas* L.) Roots

**DOI:** 10.3390/foods13223636

**Published:** 2024-11-14

**Authors:** Shan Zhao, Lingli Zhong, Xi Li, Lin Qin, Ya Zhou, Xinyu Lei, Xingguo Zheng, Keting Jin, Zhigang Pu, Xue Hou, Jun Song, Tao Lang, Cong Zhang, Junyan Feng

**Affiliations:** 1 Institute of Quality Standard and Testing Technology Research, Sichuan Academy of Agricultural Sciences, Chengdu 610066, China; zhaoshan11@126.com (S.Z.);; 2 Biotechnology and Nuclear Technology Research Institute, Sichuan Academy of Agricultural Sciences, Chengdu 610061, China

**Keywords:** sweet potato roots, flesh colors, nutrients, phytochemicals, minerals

## Abstract

Sweet potato (*Ipomoea batatas* (L.) is regarded among the most crucial crops globally because it is abundant in essential nutrients vital for human health. However, limited comprehensive information is available regarding the nutritional composition of sweet potato, which hinders its optimal utilization. This study investigated the nutritional and chemical composition of sweet potato roots and explored their interrelationships. In total, 86 sweet potato accessions, comprising white, yellow, orange, and purple flesh-colored varieties, were used. A total of 34 components, including nutrients, phytochemicals, and minerals, were identified. Multivariate analysis was performed to assess the relationships among these components. The sweet potato roots were rich in carbohydrates, polyphenols, and minerals. Carbohydrates were primarily composed of total starch (22.6–69.7 g/100 g DW), total soluble sugar (TSS) (10.3–40.0 g/100 g DW), and total dietary fiber (TDF) (7.99–26.0 g/100 g DW). Polyphenols included total caffeoylquinic acids (CQAs) (0.478–14.2 g/kg DW), total anthocyanins (0–2003 mg/kg DW), and β-carotene (0–133 mg/kg DW). The mineral content followed the order: potassium > calcium > phosphorus > sodium > magnesium > iron > manganese > zinc > copper > selenium. White-fleshed sweet potato exhibited high total starch levels (50.4 g/100 g DW) but low TSS levels (21.1 g/100 g DW). Orange-fleshed sweet potato contained high levels of TSS (26.5 g/100 g DW), TDF (17.9 g/100 g DW), and β-carotene (61.4 mg/100 g DW) but low levels of protein (2.99 g/100 g DW) and total starch (43.0 g/100 g DW). Purple-fleshed sweet potato had high levels of phytochemicals, particularly total CQAs (8.17 g/kg DW) and anthocyanins (904 mg/kg DW). Cluster analysis categorized sweet potato accessions into six clusters with unique characteristics. Furthermore, principal component analysis identified accessions with exceptionally high nutritional content. The correlation analysis indicated that starch was negatively correlated with soluble sugar and TDF, whereas CQAs and anthocyanins were highly positively correlated. These findings offer a solid theoretical foundation for sweet potato breeding and utilization.

## 1. Introduction

Sweet potato (*Ipomoea batatas* (L.) Lam.; family: Convolvulaceae) is a tuberous-rooted plant and ranks among the world’s primary staple crops. China is the largest producer of sweet potatoes, contributing to 54% of global production [1]. Its storage roots are a rich source of nutrition, containing substantial amounts of starch, sugar, protein, vitamins, and minerals. Due to its high yield and carbohydrate content, sweet potato is considered a promising crop for combating hunger in developing countries. Conversely, in developed countries, where overnutrition is a growing public health concern, sweet potato presents functional components beneficial in managing chronic conditions such as cardiovascular and intestinal diseases. These components include carotenoids, anthocyanins, caffeoylquinic acids (CQAs), dietary fiber, and resistant starch [2,3,4,5,6,7].

Globally, numerous sweet potato varieties are cultivated, which differ in skin and flesh colors, shapes, sizes, taste, and texture [8]. Research has extensively investigated the nutritional composition of these varieties, especially focusing on white-fleshed, yellow- or orange-fleshed, and purple-fleshed varieties [9,10,11,12,13]. Multivariate statistical analyses, including cluster analysis and principal component analysis (PCA), have been performed to manage complex data and assess the relationships between the biochemical characteristics of these varieties [14]. In general, white-fleshed sweet potato is known for its high starch content, orange-fleshed sweet potato is recognized for its elevated β-carotene levels, and purple-fleshed sweet potato is notable for its high anthocyanin contents [15,16].

Sweet potato holds immense promise in combating food insecurity and addressing malnutrition, undernutrition, and overnutrition across both developing and developed nations. Its unique agronomic features and rich nutritional composition make it a key crop for sustainable food systems. However, the limited comprehensive understanding of its full nutritional profile restricts its optimal exploitation. Enhancing awareness of sweet potato’s nutritional value and its potential for future economic impact is critical for improving national and global food systems.

In this study, we used 86 sweet potato accessions, representing white-fleshed sweet potato (WFSP), yellow-fleshed sweet potato (YFSP), orange-fleshed sweet potato (OFSP), and purple-fleshed sweet potato (PFSP) cultivars. The analysis investigated the diversity of nutrients, phytochemicals, and minerals in the roots of these varieties. By employing multivariate analysis, we sought to better understand the data structure and reveal patterns in the distribution of key nutritional traits. This research not only enhances our understanding of the nutritional diversity within sweet potato cultivars but also provides a solid scientific foundation for sweet potato breeding programs, consumption selection, and the broader utilization of this versatile crop. By advancing sweet potato breeding and utilization, this study contributes to the development of strategies that can help address global challenges related to food security and nutrition.

## 2. Materials and Methods

### 2.1. Plant Materials and Sample Preparation

We here used 86 sweet potato accessions, comprising 60 modern cultivars, 11 Chinese local cultivars, 12 commercially available materials, and 3 introduced Japanese cultivars. Based on their flesh colors, all accessions were segregated into 40 WFSP, 21 YFSP, 9 OFSP, and 16 PFSP (Table 1).

All the sweet potato accessions were cultivated following standard agricultural practices at the XinDu experimental station (104°12′ E, 30°46′ N) under the guidance of the Sichuan Academy of Agricultural Sciences in 2020, employing an augmented block design. Planting was performed in March, followed by transplantation in May and harvesting in November. Following harvesting, intact roots were selected for the experiment, cleaned, and chopped using the quartering method, with three replicates performed. The samples were then dried at 60 °C for 16 h. Subsequently, all the dried samples were powdered using a commercial grinder and stored at −20 °C until further analysis.

### 2.2. Reagents, Solvents, and Standards

Standard products were procured from Dr Ehrenstorfer GmbH (Augsburg, Germany) and Sigma–Aldrich Fine Chemicals (St. Louis, MO, USA). High-performance liquid chromatography (HPLC)-grade reagents were obtained from MERCK (Darmstadt, Germany) and were used to prepare the mobile phases. All other chemicals and reagents were of analytical grade or HPLC purity. Milli-Q ultrapure water was used for preparing aqueous solutions.

### 2.3. Principal Nutrients, Dietary Fiber, and Resistant Starch

Dry matter content was calculated by measuring the weight difference before and after drying. Moisture content was measured gravimetrically following the method described in the Official Methods of Analysis of the Association of Analytical Chemists [17] (AOAC method 934.01). Total dietary fiber (TDF) was analyzed using the enzymatic gravimetric method (AOAC 991.43). By employing the Kjeldahl method, protein content was measured using a Kjeltec 8200 instrument (Foss, Hillerød, Denmark), applying a nitrogen-to-protein conversion coefficient of 6.25. To estimate total starch content, the samples were extracted with 80% ethanol and centrifuged to remove the supernatant. The remaining residue was employed to assess total starch content using the MEGAZYME kit (AOAC 996.11) (Megazyme, Bray, Ireland). Amylose content was measured colorimetrically, as described by Hoover et al. [18], relying on the formation of an amylose-iodine complex. A standard calibration curve was prepared using potato amylose purchased from Sigma–Aldrich (St. Louis, MO, USA). The amylose content was expressed as a ratio of amylose to total starch. Resistant starch was quantified using the MEGAZYME kit following the approved method AACC 32-40.01 [19].

### 2.4. Soluble Sugars

Soluble sugars were quantified through HPLC following Galvao et al.’s method [20]. The specific HPLC conditions are detailed in Supplementary Method S1. Fructose, glucose, and sucrose were used as quantitative reference standards, with results expressed in grams per 100 g of dry weight (g/100 g DW), and the limit of quantitation (LOQ) was 0.2 g/100 g.

### 2.5. β-Carotene Quantification

β-Carotene was measured following Hosotani et al.’s procedure [21], with some modifications. Briefly, 200 mg of the sample was mixed with water (3 mL), ascorbic acid (200 mg), and α-amylase (100 mg). After the samples were incubated at 55 °C for 30 min, they were extracted with 15 mL ethanol and oscillated at 60 °C for 30 min. Then, 100% (*m*/*v*) KOH (5 mL) was added to the samples, and the mixture was saponified at 53 °C for 30 min. After the mixture was cooled, the samples were extracted twice with petroleum ether (15 mL) and gently shaken for 10 min. This step was repeated once. Then, the organic phase was washed with water until it was neutral. The petroleum ether extracts were dried under N2 at 40 °C. The residue was dissolved in 5 mL ethanol for HPLC analysis, as detailed in Supplementary Method S2. β-Carotene was used as the standard, and the external standard method was applied for quantitative analysis. Results were expressed as milligrams per kilogram of dry weight (mg/kg DW). The LOQ was 0.1 mg/kg.

### 2.6. Determination of Caffeoylquinic Acids

CQAs were extracted by weighing 200 mg of the sample into a 20 mL centrifuge tube, followed by the addition of 10 mL of a 70% *v*/*v* methanol aqueous solution. The tubes were immersed in a water bath at 65 °C and sonicated for 30 min at an oscillating frequency of 40 kHz. The mixture was then centrifuged, and the supernatant was transferred to a 25 mL brown volumetric flask. This step was repeated once to ensure complete extraction, and the final volume was adjusted to 25 mL. The extract was then filtered through a 0.22-μm nylon membrane before the HPLC analysis (Supplementary Method S3). The specific CQAs quantified included caffeic acid, 3-O-Caffeoylquinic acid (3-CQA), 4-O-caffeoylquinic acid (4-CQA), 5-O-caffeoylquinic acid (5-CQA), 3,4-di-O-caffeoylquinic acid (3,4-diCQA), 3,5-di-O-caffeoylquinic acid (3,5-diCQA), 4,5-di-O-caffeoylquinic acid (4,5-diCQA), and 3,4,5-tri-O-caffeoylquinic acid (3,4,5-triCQA). The results were expressed as grams per kilogram of dry weight (g/kg DW). The LOQ was 0.02 g/kg.

### 2.7. Determination of Anthocyanins

Acid hydrolysis of anthocyanins was performed following a previous procedure with some modifications [22]. A 500 mg sample was weighed, and 10 mL of an aqueous solution consisting of absolute ethanol, water, and hydrochloric acid (37%, *w*/*v*) at a 2:1:1 ratio (*v*/*v*/*v*) was added. The mixture was sonicated for 30 min at an oscillating frequency of 40 kHz. Then, anthocyanin hydrolysis was conducted under a nitrogen atmosphere for 60 min at 100 °C. The hydrolysate was then cooled in an ice bath, and the extract was filtered using a 0.22-µm polytetrafluoroethylene membrane. The hydrolyzed anthocyanins were subsequently analyzed through HPLC, with specific parameters detailed in Supplementary Method S4. Peonidin, cyanidin, and pelargonidin served as standard reference materials for quantitative determination, with results presented as milligrams per kilogram of dry weight (mg/kg DW), with a LOQ established at 1.0 mg/kg.

### 2.8. Mineral Content

Minerals analysis was conducted following a previous procedure with some modifications [23]. A 0.5 g sample was placed in a Teflon^®^ (PTFE) digestion tube, and 7 mL of 65% HNO3 was added. The mixture was digested in a microwave oven (TOPEX, PreeKem, Shanghai, China) at 200 °C for 25 min. Following digestion, the solution was diluted with ultrapure water. The concentrations of 10 elements, namely iron, copper, zinc, sodium, potassium, calcium, magnesium, manganese, phosphorous, and selenium, were measured through inductively coupled plasma mass spectrometry (NexION 300D, PerkinElmer, Waltham, MA, USA). Instrument conditions are detailed in Supplementary Method S5. Results for sodium, potassium, calcium, magnesium, iron, and phosphorous were expressed as grams per kilogram of dry weight (g/kg DW), while manganese, copper, zinc, and selenium were presented as milligrams per kilogram of dry weight (mg/kg DW). The LOQs were set at 0.02 mg/kg for selenium, 0.3 mg/kg for copper and manganese, and 3 mg/kg for other elements.

### 2.9. Quality Control

The results were expressed as mean ± standard deviation (SD), with all measurements conducted in triplicate. Market sweet potato flour, which underwent repeated analysis alongside certified reference materials, was utilized as an in-house quality control material for all nutrient analyses in every batch. In the minerals analysis, reagent blanks and reference materials were processed simultaneously with the samples. The certified reference material of sweet potato flour (GBW10199, TMRM Quality Inspection Technology Co., Ltd., Changzhou, China) was employed to calculate recovery and accuracy. The recovery rates for all minerals fell within the acceptable limits of the certified range for elemental concentrations and relative standard deviations.

### 2.10. Statistical Analysis

Data were analyzed using SPSS 22.0 software (SPSS, Inc., Chicago, IL, USA). Differences among various nutritional parameters were evaluated using a one-way analysis of variance, followed by LSD multiple comparisons to assess mean differences at a significance level of *p* < 0.05. Pearson’s correlation coefficient was calculated to illustrate the relationships between nutritional parameters, with correlation coefficients categorized as follows: very strong correlation (r = 0.90–1.0), strong correlation (r = 0.70–0.89), moderate correlation (r = 0.50–0.69), weak correlation (r = 0.30–0.49), and negligible correlation (r < 0.30) [24]. The PCA was performed to reduce dataset dimensionality and describe variability within the system using a smaller number of variables. The first principal component (PC) captured the maximum possible variation, while subsequent components accounted for the next highest variations. The unsupervised classification was conducted using cluster analysis to measure the similarity between objects. A dendrogram was constructed using Ward’s clustering algorithm, employing squared Euclidean distance as the distance metric and linkage method for clusters. A heatmap illustrating relative composition and Pearson’s correlation coefficients was generated using a web interface (https://www.chiplot.online/) (accessed on 18 November 2022).

## 3. Results

### 3.1. Nutrient Analysis of Sweet Potato Roots

Table 2 and Table S1 present the nutrient composition of 86 sweet potato accessions. The average dry matter content was 25.2 g/100 g (range: 18.0–36.5 g/100 g). The protein content varied significantly from 2.15 g/100 g DW in Funingshu12 to 7.11 g/100 g DW in Sanguishu15 (mean: 3.55 g/100 g DW). Among the components of sweet potato roots, starch, soluble sugars, and dietary fiber were the three most abundant. Total starch content ranged from 22.6 to 69.7 g/100 g DW (mean: 48.5 g/100 g DW). Notably, 40 accessions (almost half) exhibited total starch levels exceeding 50 g/100 g DW, and 4 accessions surpassed 60 g/100 g DW: Jishu8088, 2-190, CA9, and Baiguqilong. Amylose content varied between 15.3% and 34.7% (mean: 23.2%). The coefficient of variation (CV) of resistant starch was 102% (range: 0.254–9.12 g/100 g DW; mean: 1.29 g/100 g DW). Only 14 cultivars had resistant starch content of more than 2 g/100 g DW. HPLC detection revealed that total soluble sugars (TSS) primarily consisted of fructose, glucose, and sucrose, with sucrose representing the highest content in sweet potato roots. The mean TSS content was 22.3 g/100 g DW (range: 10.3–40.0 g/100 g DW). CA2 exhibited the highest TSS content (40 g/100 g DW), while all other accessions had TSS contents ≤ 30 g/100 g DW. Most accessions (59 in total) had TSS values between 20 and 30 g/100 g DW. Fructose and glucose exhibited high CVs at 53.0% and 55.5%, respectively. Fructose had a mean value of 3.53 g/100 g DW (range: 0.51–10.6 g/100 g DW), and glucose had a mean value of 4.08 g/100 g DW (range: 0.56–10.6 g/100 g DW). Sucrose had a mean value of 14.7 g/100 g DW (range: 2.75–36.2 g/100 g DW). The TDF content varied between 7.99 and 26.0 g/100 g DW (mean: 16.9 g/100 g DW). Twelve accessions had a TDF content of >20 g/100 g DW, while 52 accessions fell within the range of 15–20 g/100 g DW.

Phytochemical variability in sweet potato roots showed substantial CVs, ranging from 56.4% to 288%, particularly for anthocyanins and β-carotene. Total anthocyanins, comprising cyanidin, paeoniflorin, and pelargonidin (predominantly cyanidin and paeoniflorin), were found exclusively in PFSPs. Across all accessions, total anthocyanins ranged from 0 to 2003 mg/kg DW, with a mean of 904 mg/kg DW in PFSPs (range: 322–2003 mg/kg DW). For β-carotene, the mean value was 10.5 mg/kg DW (range: 0–133 mg/kg DW). Seven accessions exhibited β-carotene content of >30 mg/kg DW, with Pushu32 (130 mg/kg DW) and Chuanshu20 (115 mg/kg DW) standing out. Eight free phenolic acids were also analyzed, including 5-CQA, 3-CQA, 4-CQA, 3,4-diCQA, 3,5-diCQA, 4,5-diCQA, 3,4,5-triCQA, and caffeic acid. The CVs for these phenolic acids were notable, ranging from 56.4% to 112%. Total CQAs ranged from 0.478 g/kg DW to 14.2 g/kg DW (mean: 4.16 g/kg DW). The phenolic acids 3-CQA (0.0922–5.69 g/kg DW), 3,5-diCQA (0.131–4.04 g/kg DW), 3,4-diCQA (0.0536–2.02 g/kg DW), and 4,5-diCQA (0.0516–1.37 g/kg DW) were particularly abundant. Eighteen accessions had total CQAs of >6 g/kg DW, of which 11 were PFSPs.

The mineral content of sweet potato roots demonstrated moderate variability, with relatively low CVs ranging from 21.6% to 49.9%. Potassium was the most abundant mineral (average: 12.4 g/kg DW, with a range of 5.56 g/kg DW (3-14-52) to 24.8 g/kg DW (ChuanH5). Sodium content varied between 0.492 g/kg DW and 4.95 g/kg DW (mean: 1.68 g/kg DW). The average calcium content was 2.56 g/kg DW (range: 0.638–6.33 g/kg DW), with SH5 having the highest calcium concentration. Magnesium levels ranged from 0.373 g/kg DW to 2.16 g/kg DW (mean: 1.22 g/kg DW). Phosphorus content had the lowest CV among the minerals (mean: 2.42 g/kg DW; range: 1.32–4.40 g/kg DW). Iron levels varied from 0.0731 g/kg DW to 0.593 g/kg DW (mean: 0.186 g/kg DW). Copper, zinc, and manganese showed mean values of 5.29, 9.11, and 15.0 mg/kg DW, respectively (content range: 3.03–10.4, 5.69–17.6, and 3.72–37.3 mg/kg DW, respectively). Selenium content was relatively low (range: 0.0294–0.247 mg/kg DW; mean: 0.0831 mg/kg DW).

### 3.2. Comparative Analysis of Nutrients in Colored Roots

**Figure 1:**
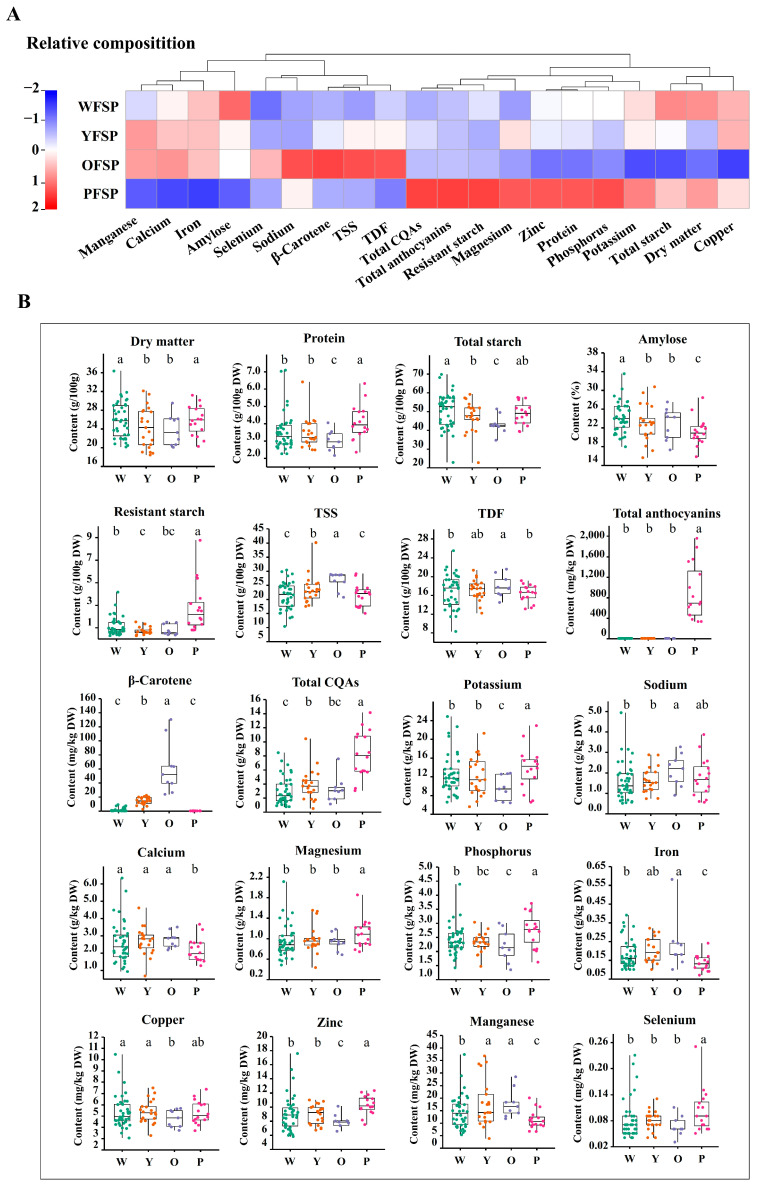
Comparative analysis of the content of nutrients, phytochemicals, and minerals in colored sweet potato roots. (**A**): Heat map of the relative mean values of nutritional composition in different flesh-colored sweet potato roots. (**B**): Box plot depiction of the distribution of the content of each nutritional composition in different flesh-colored sweet potato roots. WFSP/W: white-fleshed sweet potato; YFSP/Y: yellow-fleshed sweet potato; OFSP/O: orange-fleshed sweet potato; PFSP/P: purple-fleshed sweet potato. Different letters indicate statistical significance determined using LSD multiple comparisons (*p* <  0.05). TSS: total soluble sugar; TDF: total dietary fiber; CQAs: caffeoylquinic acids.

The nutrient content among sweet potato roots of different flesh colors was compared (Figure 1 and Table S2). WFSPs had the highest average content of dry matter (25.8 g/100 g), total starch (50.4 g/100 g DW), and amylose (24.3%), with the amylose content being significantly higher than that in the other flesh-colored sweet potatoes (*p* < 0.05). However, WFSPs exhibited the lowest average levels of TSS (21.1 g/100 g DW), total CQAs (2.94 g/kg DW), as well as TDF and β-carotene. OFSPs were characterized by high levels of mean TSS (26.5 g/100 g DW), TDF (17.9 g/100 g DW), β-carotene (61.4 g/100 g DW), and sodium (2.11 g/kg DW). However, they exhibited lower average levels of protein (2.99 g/100 g DW), total starch (43.0 g/100 g DW), potassium (9.49 g/kg DW), phosphorus (2.19 g/kg DW), copper (4.77 mg/kg DW), and zinc (7.96 mg/kg DW). PFSPs exhibited higher average levels of dry matter (25.7 g/100 g), protein (4.08 g/100 g DW), total starch (48.8 g/100 g DW), resistant starch (2.74 g/100 g DW), total CQAs (8.17 g/kg DW), potassium (13.8 g/kg DW), magnesium (1.10 g/kg DW), phosphorus (2.75 g/kg DW), zinc (10.3 mg/kg DW), and selenium (0.102 mg/kg DW) than the other flesh-colored sweet potatoes. By contrast, they had lower average levels of amylose (21.2%), TSS (21.4 g/100 g DW), TDF (16.4 g/100 g DW), calcium (2.16 g/kg DW), iron (0.134 g/kg DW), and manganese (11.4 mg/kg DW). Importantly, total CQAs were more than twice as high in PFSPs compared with the other colored cultivars. Anthocyanins were exclusively found in PFSPs. Compared with other flesh-colored sweet potatoes, YFSPs had average nutrient levels between OFSPs and WFSPs, without being notably high or low in any specific component.

### 3.3. Cluster Analysis

**Figure 2:**
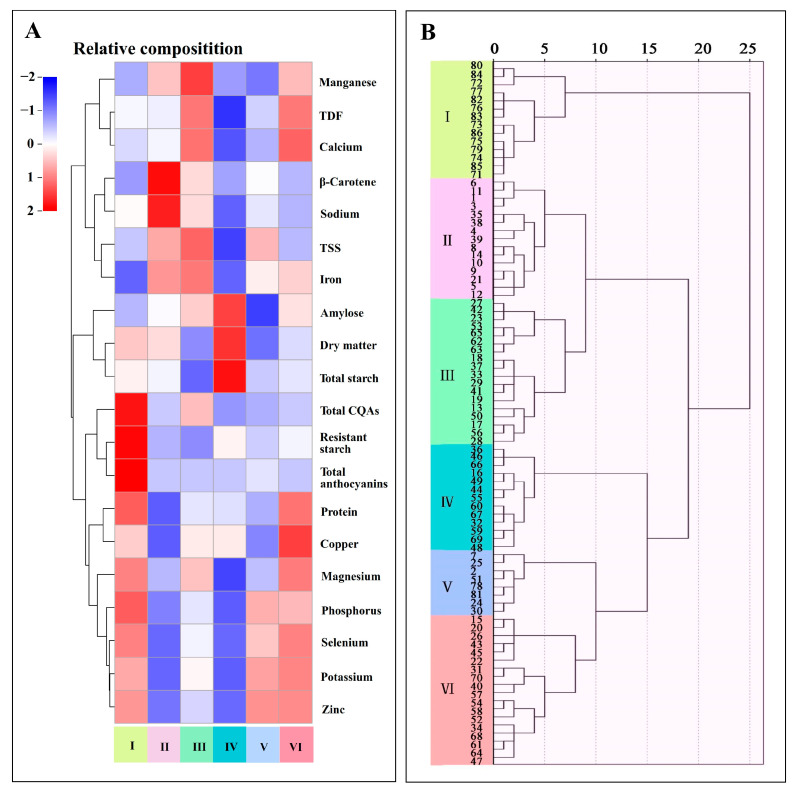
Cluster analyses of sweet potato accessions. (**A**): Heat map of the relative mean values of nutritional composition in each cluster cultivar. (**B**): Dendrogram of cluster analysis in 86 accessions.

The cluster analysis was performed to assess the multivariate relationship among the biochemical attributes. The analysis effectively grouped the sweet potato accessions into six hierarchical clusters (depicted in Figure 2B through a dendrogram). The unique nutrient profiles of the six clusters were visualized using a heatmap (Figure 2A and Table S3).

Cluster I, which consisted 14 accessions, was characterized by considerably high levels of protein (4.11 g/100 g DW), resistant starch (2.83 g/100 g DW), total anthocyanins (983 ± 531 mg/kg DW), total CQAs (8.88 g/kg DW), potassium (13.8 g/kg DW), magnesium (1.14 g/kg DW), phosphorus (2.75 g/kg DW), zinc (10.2 mg/kg DW), and selenium (0.0971 mg/kg DW), but low levels of iron (0.125 g/kg DW) and β-carotene (0 mg/kg DW). Cluster II, which consisted of 15 accessions, was notable for high levels of β-carotene (33.9 ± 33.6 mg/kg DW), sodium (2.19 g/kg DW), and iron (0.217 g/kg DW) but low levels of protein (2.91 g/100 g DW), potassium (9.90 g/kg DW), phosphorus (2.17 g/kg DW), copper (4.64 mg/kg DW), zinc (7.74 mg/kg DW), and selenium (0.0649 mg/kg DW). Cluster III, which consists of 18 accessions, exhibited high levels of TSS (26.0 g/100 g DW), TDF (18.7 g/100 g DW), calcium (3.12 g/kg DW), iron (0.230 g/kg DW), and manganese (20.4 mg/kg DW), whereas low levels of dry matter (22.7 g/100 g), total starch (42.2 g/100 g DW), and resistant starch (0.580 g/100 g DW). Cluster IV, which had 13 accessions, was distinguished by considerably high levels of dry matter (29.4 g/100 g), total starch (58.9 g/100 g DW), and amylose (26.8%), whereas low levels of other constituents. Cluster V, which comprised 8 accessions, was defined by high levels of potassium (13.9 g/kg DW), phosphorus (2.59 g/kg DW), zinc (10.3 mg/kg DW), and selenium (0.0930 mg/kg DW), whereas low levels of dry matter (22.1 g/100 g), total starch (46.4 g/100 g DW), amylose (19.0%), and manganese (9.93 mg/kg DW). Cluster VI, which had 18 accessions, was characterized by high levels of protein (4.03 g/100 g DW), TDF (18.7 g/100 g DW), potassium (14.4 g/kg DW), calcium (3.19 g/kg DW), magnesium (1.15 g/kg DW), phosphorus (2.57 g/kg DW), copper (5.93 mg/kg DW), zinc (10.3 mg/kg DW), manganese (16.5 mg/kg DW), and selenium (0.103 mg/kg DW), whereas low levels of sodium (1.49 g/kg DW). PFSPs were prominently found in cluster I, which exhibited the highest anthocyanin content. The other clusters showed negligible or minimal anthocyanin content, reflecting the significant biochemical differences among the sweet potato accessions based on their flesh color and nutrient composition.

### 3.4. Principal Component Analysis

**Figure 3:**
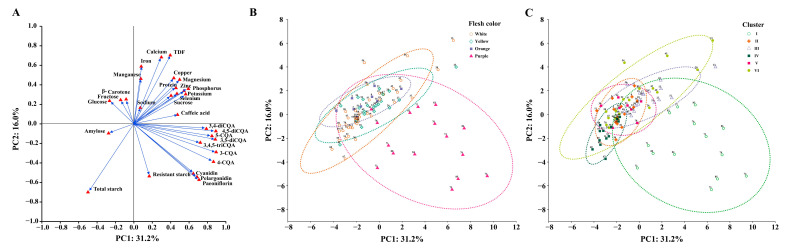
Two-dimensional PCA score plots on PC1 and PC2. (**A**): Factor loading plots of nutritional traits; (**B**): PCA score plots grouped by flesh color; (**C**): PCA score plots grouped by clusters.

To explore the factors contributing to the variability and relationships among these parameters, PCA was applied to the standardized nutritional parameters of the 86 sweet potato accessions. The PCA results were interpreted based on the interrelationships among varying components. PCA was performed on proteins, total starch, resistant starch, amylose, fructose, glucose, sucrose, TDF, β-carotene, cyanidin, paeoniflorin, pelargonidin, CQAs, caffeic acid, and minerals. The Bartlett test yielded a significance level of *p* < 0.001, and the Kaiser-Meyer-Olkin (KMO) value was 0.751, confirming the adequacy of the correlation matrix for PCA. The first seven PCs collectively accounted for 78.0% of the variability among the accessions, with PC1, PC2, PC3, PC4, PC5, PC6, and PC7 explaining 31.2%, 16.0%, 9.39%, 7.34%, 6.22%, 4.16%, and 3.81% of the total variance, respectively (Table S4). Positive factor loadings (FL) suggest that the specific factor will be higher on the positive axis. Cyanidin (FL1: 0.648), paeoniflorin (FL1: 0.710), pelargonidin (FL1: 0.688), CQAs (FL1: 0.724–0.896), TDF (FL2: 0.701), calcium (FL2: 0.681), fructose (FL3: 0.840), and glucose (FL3: 0.849) had large positive FL values. Correspondingly, negative FL values determine that the specific factor will be higher on the negative axis. Total starch (FL2: −0.702) had a negative FL value (Figure 3A).

In the PCA, loading reflects the contribution of each nutritional variable to a specific PC, and uniqueness represents the proportion of variation that is specific to a variable and not captured by PCs. The aforementioned dominant factors displayed high component loading and low uniqueness. A variable with a high loading and low uniqueness significantly contributes to the PCA, indicating that much of its variation is explained by the PCs. The two-dimensional PCA score plots were used to illustrate the distribution of nutritional traits across the 86 sweet potato accessions. PC1 and PC2 scores were plotted according to flesh color and clustering, respectively, and enabled visualization of the variability according to these parameters (Figure 3B,C). The position of accessions on the PCA plot can be interpreted in terms of their nutritional profiles. For example, in the southeast direction, accessions Xuzishu9 (74), Xuzishu8088 (75), and Rizishu9 (85) were identified as having high phytochemical and starch content, making them nutritionally distinct. In the northeast direction, accessions ChuanH5 (15), Yongchunwuchi (20), and CA2 (23) were characterized by exceptionally low starch content. Notably, CA2 also had exceptionally high sucrose content, indicating a unique carbohydrate profile. In the northwestern direction, Sanguishu15 (54) stood out with its particularly high protein content, positioning it as a protein-rich variant. In the southwestern direction, accessions 2-190 (60) and CA9 (67) exhibited high starch content, suggesting their role as starch-dense sweet potato varieties.

### 3.5. Correlation Analysisamongnutrients, Phytochemicals, and Minerals

**Figure 4:**
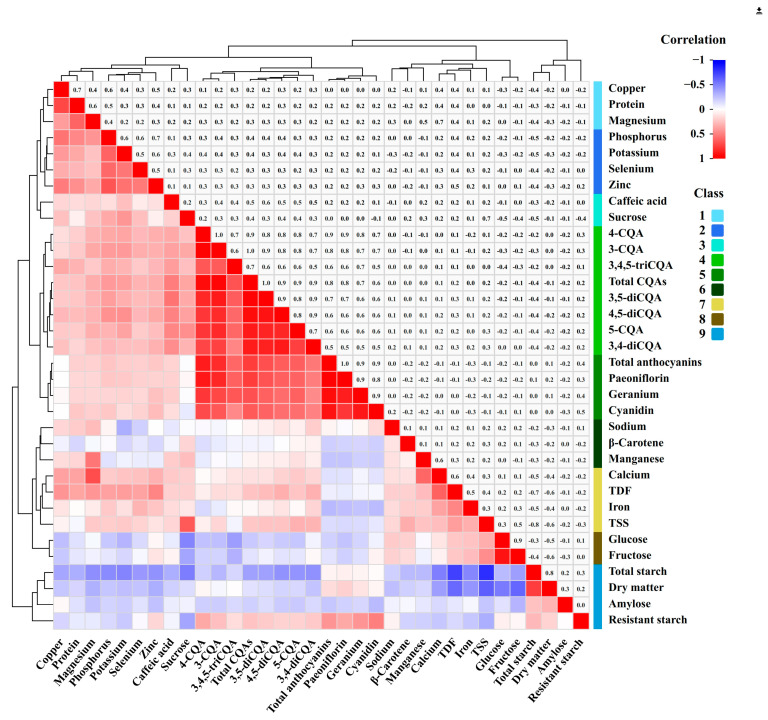
Heat map of Pearson’s correlational analysis. The parameters were clustered and labeled into 9 classes by color.

The correlation analysis among the 34 nutritional factors revealed several significant relationships across nutrients (10), phytochemicals (14), and minerals (10) (Figure 4 and Table S5).

Dry matter was strongly positively correlated with total starch, whereas it weakly positively correlated with amylose. It exhibited a moderate negative correlation with fructose, glucose, TSS, and TDF, with a weak negative correlation with calcium, magnesium, iron, and zinc. Protein was strongly positively related to copper, moderately positively related to magnesium, weakly positively related to TDF, potassium, calcium, phosphorus, and zinc, and weakly negatively related to total starch. Total starch exhibited strong negative correlations with TSS and TDF, moderate negative correlations with sucrose and calcium, and weak negative correlations with fructose, 5-CQA, caffeic acid, 3,4-diCQA, 3,5-diCQA, 4,5-diCQA, total CQAs, potassium, magnesium, phosphorus, iron, copper, zinc, and selenium. Resistant starch exhibited a moderate positive correlation with cyanidin, a weak positive correlation with paeoniflorin, pelargonidin, total anthocyanins, and 4-CQA, and a weak negative correlation with sucrose and TSS. Fructose and glucose exhibited a strong correlation, with a correlation coefficient of 0.900 (*p* < 0.01). Consequently, fructose and glucose were more similarly correlated with other components. They were weakly positively correlated with TSS, whereas weakly negatively correlated with sucrose and 3,4,5-triCQA. Sucrose exhibited a moderate positive correlation with TSS and a weak positive correlation with 5-CQA, 3-CQA, 3,4-diCQA, 3,5-diCQA, 4,5-diCQA, total CQAs, potassium, and phosphorus. TSS was weakly positively correlated with TDF, β-carotene, 5-CQA, and iron. TDF was moderately positively correlated with calcium, weakly positively correlated with other minerals (except sodium and manganese), and weakly positively correlated with 3,4-diCQA and 4,5-diCQA.

Cyanidin, paeoniflorin, pelargonidin, total anthocyanins, 5-CQA, 3-CQA, 4-CQA, 3,4-diCQA, 3,5-diCQA, 4,5-diCQA, 3,4,5-triCQA, and total CQAs were all positively correlated with one another, and their correlation coefficients ranged from 0.395 (*p* < 0.01) to 0.979 (*p* < 0.01). Total anthocyanin was strongly positively correlated (r > 0.9, *p* < 0.01) with cyanidin, paeoniflorin, and pelargonidin. A strong positive correlation was observed between total CQAs and 3-CQA, 4-CQA, 3,5-diCQA, and 4,5-diCQA; between 3-CQA and 4-CQA; and between 3,5-diCQA and 4,5-diCQA. 5-CQA, 3-CQA, 4-CQA, 3,5-diCQA, 4,5-diCQA. Total CQAs were weakly positively correlated with potassium, magnesium, phosphorus, and zinc. A weakly positive correlation was observed between 3-CQA and selenium. 3,4-diCQA and 3,4,5-triCQA exhibited weak positive correlations with zinc, potassium, phosphorus, and copper, respectively.

Potassium was moderately positively correlated with phosphorus, zinc, and selenium, whereas negatively weakly correlated with sodium. Calcium exhibited a moderate positive correlation with magnesium and manganese and a weak positive correlation with iron, copper, and zinc. Magnesium exhibited a moderate positive correlation with manganese and a weak positive correlation with phosphorus, copper, and zinc. A moderate positive correlation was observed between phosphorus and copper, zinc, and selenium. Iron and selenium were weakly positively correlated. Copper showed a moderately positive correlation with zinc and a weakly positive correlation with selenium. Zinc and selenium were moderately positively correlated.

## 4. Discussion

### 4.1. Nutrient Composition of Sweet Potato Roots

Sweet potato is recognized as a significant food source because of its rich content of carbohydrates, minerals, and phytochemicals. Studies have highlighted the variability in the nutritional composition (such as dry matter, protein, starch, sugar, and dietary fiber content) of sweet potatoes across different varieties, emphasizing their adaptability and nutritional potential. For instance, Oboh et al. [25] analyzed 49 varieties of sweet potato, finding significant variation in dry matter (17.8%–36.8%) and protein (1.4%–9.4%). Similarly, Senanayake et al. [26] evaluated five Sri Lankan sweet potato tuber flours, reporting starch content between 33% and 64%, crude protein ranging from 1.2% to 3.3%, and amylose content from 15.4% to 19.6%. Cui et al. [27] investigated seven New Zealand sweet potato varieties and identified starch contents of 487–719 g/kg, protein contents of 32.5–57.5 g/kg, and total dietary fiber contents of 37.0–99.0 g/kg. Their analysis also showed significant variation in sugar content, with fructose ranging from 25.5 to 81.5 g/kg, glucose from 4.3 to 102.1 g/kg, and sucrose from 25.2 to 163.0 g/kg. The study findings are consistent with those of earlier studies in terms of nutritional content, especially about the high carbohydrate content in sweet potato roots, which ranged from 51.5% to 81.6% in this study. However, some studies on carbohydrates also included dietary fiber in the carbohydrate measurement [28], and with the incorporation of TDF content, the carbohydrate content increased to 77.0%–94.4%. These results highlight sweet potato’s versatility as a valuable crop, capable of addressing hunger by providing a rich source of energy.

The anthocyanin profile of PFSPs is notably complex because of the presence of multiple anthocyanin compounds, which include non-acylated, mono-acylated, and diacylated glucosides of cyanidin, peonidin, and pelargonidin [29]. Acid hydrolysis is often used to detach the glycons from the flavylium ion of anthocyanin compounds, releasing key anthocyanidins such as cyanidin, delphinidin, malvidin, peonidin, pelargonidin, and petunidin [22,30]. This process converts anthocyanins into their monomeric forms, facilitating more precise analysis. Truong et al. [22] found that PFSP genotypes contained between 828 and 1992 mg/100 g DW of total phenolics and between 3 and 328 mg/100 g DW of total monomeric anthocyanins. Similarly, Rumbaoa et al. [31] reported that the total root phenolic content in five Philippine-grown sweet potato cultivars ranged from 192.7 to 1159.0 mg GAE/100 g, further underscoring the high phytochemical content of PFSP roots. These rich levels of anthocyanins and phenolics highlight PFSP’s exceptional nutritional and health-promoting properties.

Regarding mineral element contents, sweet potato roots are also highly nutritious, with their mineral composition ranked as follows: potassium > calcium > phosphorus > sodium > magnesium > iron > manganese > zinc > copper > selenium. The relatively high levels of potassium, sodium, calcium, and iron make sweet potato roots a valuable dietary source for mineral supplementation. However, studies have reported that variations in soil and fertilizer management practices can lead to differences in the mineral content of the roots [32].

Variations in the nutritional composition of sweet potato roots can be attributed to several factors, including differences in planting environments, harvest times, and sample preparation methods. To minimize these sources of variation and ensure comparability across the accessions in this study, all sweet potato materials were sown, transplanted, and harvested simultaneously, whereas growth conditions, sample preparation procedures, and storage conditions were kept consistent across the accessions. Thus, we believe that the different accessions were comparable. This careful control of experimental conditions allows for more reliable comparisons among the accessions. However, due to the logistical challenges of analyzing a large number of accessions and parameters using fresh samples, the fresh sweet potato roots were dried and converted into powder before conducting the analysis. The drying process may induce changes in thermally unstable components, such as β-carotene, phenolic acid, and anthocyanins [33]. Consequently, the measured concentrations of these components in the study may deviate from those found in fresh samples, potentially affecting their reported values.

### 4.2. Comparative Analysis of Nutrients in Different Flesh-Colored Roots

Sweet potato roots exhibit unique nutritional profiles that are intricately linked to their flesh color. Franková et al. [34] indicated that PFSPs have significantly higher total phenolic content than OFSPs, whereas carotenoid levels are markedly greater in OFSPs. Furthermore, phenolic acids and flavonoids are relatively more abundant in the PFSPs than in the OFSP and WFSP varieties [35]. Shekhar et al. [36] highlighted that WFSPs have a high percentage of carbohydrates, reducing sugars, and phenolics, whereas OFSPs are characterized by increased levels of total protein, flavonoids, anthocyanins, and carotenoids. This study investigated the differences in nutrient content among various sweet potato accessions based on their flesh color. WFSPs were distinguished by high starch and amylose content but exhibited low levels of soluble sugars. OFSPs, on the other hand, contained high amounts of soluble sugars, dietary fiber, and β-carotene, along with lower protein and starch content. They were also rich in minerals such as calcium and manganese. PFSPs were characterized by high concentrations of phytochemicals such as polyphenols and anthocyanins, as well as elevated levels of protein, starch, and resistant starch. Conversely, they had low soluble sugar content but were rich in minerals such as zinc, phosphorus, and potassium. Overall, some of these findings corroborate previous research, yet the data presented here offer a more comprehensive analysis of nutritional differences among sweet potato accessions of varying flesh colors. According to recent studies, variations in phytochemical content among different sweet potato varieties are linked to differences in the regulation and expression of genes associated with the flavonoid pathway, including transcription factors that promote anthocyanin production [37].

### 4.3. Comprehensive Evaluation and Utilization of Sweetpotato Accessions

Repositioning sweet potato as a versatile crop in the market and capitalizing on its potential for value-added products could significantly enhance its role in human food systems.

Based on the clustering analysis of various sweet potato accessions, each cluster offers unique nutritional profiles that can be targeted toward different applications. Cluster I contained accessions with high levels of functional ingredients, including phenolic acids, anthocyanins, and resistant starch. These accessions are ideal for developing functional foods or products that could benefit cardiovascular and intestinal health. Clusters II and III were characterized by high levels of soluble sugars, dietary fiber, and β-carotene, with low levels of starch. These accessions are well-suited for use as fresh food—nutritious and appetizing options that could cater to health-conscious consumers. Cluster IV accessions had high starch and dry matter content, making them perfect for processing into starch-based products, such as vermicelli, which is highly consumed in China. Additionally, these starch-rich sweet potato varieties could serve as an efficient feedstock for ethanol production, addressing the rising demand for biofuels. Clusters V and VI exhibited moderate levels of starch, soluble sugars, dietary fiber, and higher mineral content, offering the potential for diversified food processing. Through PCA, materials with exceptional characteristics were identified, including those with high levels of bioactive compounds and starch (Xuzishu9, Xuzishu8088, and Rizishu9), high starch content (2-190 and CA9), high protein content (Sanguishu15), and ultra-low starch content (ChuanH5, Yongchunwuchi, and CA2). By understanding these diverse quality characteristics, breeders and food processors can target specific sweet potato varieties for breeding programs and value-added product development.

### 4.4. Correlation Study Among Nutrients, Phytochemicals, and Minerals

Studies have demonstrated a negative correlation between β-carotene content and dry matter and starch content in sweet potato storage roots. This observation can be explained by the role of phytoene synthase genes, which are involved in carotenoid biosynthesis. Quantitative trait loci (QTLs) and gene expression analyses have shown that these genes are physically linked to sucrose synthase. The co-interaction of primary-effect QTLs affects both starch biosynthesis in amyloplasts and carotenoid accumulation, creating an inverse relationship between starch and β-carotene levels [38]. It possibly explains the correlation between dry matter, starch, and β-carotene levels in sweet potato roots, consistent with the study findings. Because sucrose and other sugars serve as substrates for starch synthesis, the enzymes sucrose synthase and UDP-glucose pyrophosphorylase catalyze the sugar-to-starch conversion step [39]. This could explain the observed inverse relationship between starch and sugar content in sweet potatoes.

Protein content in sweet potato roots was positively correlated with certain minerals such as magnesium, phosphorus, copper, and zinc. These minerals are integral as constituents and cofactors in various enzymatic proteins [40,41], which may explain their association with protein levels. Similarly, the correlation between minerals and TDF content could be due to the role of minerals as cofactors in fiber synthase enzymes, contributing to fiber biosynthesis.

Regarding phenolic compounds, the biosynthesis of phenolic acids in sweet potatoes occurs via the shikimate pathway [42,43]. Because flavonoids and anthocyanins are also derived from similar biochemical pathways, their strong positive correlation with phenolic acids can be explained. Additionally, the CQA family, which consists of isomers such as 3-CQA, 4-CQA, 5-CQA,3,4-diCQA, 3,5-diCQA, 4,5-diCQA, and 3,4,5-triCQA [44], shows a strong correlation between different isomers, as they are structurally related and often produced together in the plant.

## 5. Conclusions

Sweet potato storage roots are recognized for their significant nutritional value, containing abundant carbohydrates, phytochemicals, and minerals. However, the nutrient composition varies considerably depending on the flesh color of the sweet potato cultivars. WFSPs tend to have higher starch and amylose content but lower levels of soluble sugars. On the other hand, OFSPs are rich in soluble sugars, dietary fiber, β-carotene, calcium, and manganese but lower in protein and starch. PFSPs boast high concentrations of phytochemicals such as CQAs and anthocyanins, as well as significant amounts of protein, starch, zinc, potassium, and phosphorus, though they are deficient in soluble sugars. These findings offer a scientific foundation for selecting sweet potato varieties based on their flesh color to match specific consumption and processing requirements. Through cluster analysis, sweet potato accessions were grouped into clusters with distinct uses according to their nutritional profiles. Based on the PCA scores, accessions with high levels of certain nutrients were effectively identified. The correlation analysis revealed that dry matter and starch were strongly correlated; starch was highly negatively correlated with soluble sugars and dietary fiber, and CQAs and anthocyanins exhibited a strong positive correlation. Additionally, protein and dietary fiber were positively correlated with several essential minerals. Overall, this study provides a comprehensive understanding of the nutrient composition, phytochemicals, and mineral content in sweet potato roots. It also highlights the interrelationships between these components, offering a valuable theoretical basis for high-quality genetic breeding programs aimed at improving sweet potato varieties. Moreover, it introduces new insights into the utilization of sweet potatoes.

## Figures and Tables

**Table 1:** Material Information of 86 Accessions.

No.	Accession	Flesh Color	Origin	Type
1	Zheshu851	Orange	China	Modern cultivar
2	Huang16	White	China	Local cultivar
3	Jihongrouhong	Orange	China	Local cultivar
4	Xiangshu15	White	China	Modern cultivar
5	CA8	Orange	China	Commercially available
6	Longshu9	Orange	China	Modern cultivar
7	Quanshu830	Yellow	China	Modern cultivar
8	Pushu32	Orange	China	Modern cultivar
9	Fushu604	Yellow	China	Modern cultivar
10	Funingshu12	Orange	China	Modern cultivar
11	Chuancaishu211	Orange	China	Modern cultivar
12	Beijing553	Yellow	China	Modern cultivar
13	Chuanshu20	Orange	China	Modern cultivar
14	Quanshu19	Orange	China	Modern cultivar
15	ChuanH5	White	China	Local cultivar
16	Jishu6-8	White	China	Modern cultivar
17	Chao1li	White	China	Modern cultivar
18	Fushu23	White	China	Modern cultivar
19	Jishu25	White	China	Modern cultivar
20	Yongchunwuchi	White	China	Local cultivar
21	Shengnan	Yellow	China	Modern cultivar
22	Fushu76	Yellow	China	Modern cultivar
23	CA2	Yellow	China	Commercially available
24	Chaoshu1	Yellow	China	Modern cultivar
25	Guangcaishu5	Yellow	China	Modern cultivar
26	Jishu19-40	Yellow	China	Modern cultivar
27	Fushu22	Yellow	China	Modern cultivar
28	Quanshu17	Yellow	China	Modern cultivar
29	Fushu18	Yellow	China	Modern cultivar
30	Guangcaishu2	Yellow	China	Modern cultivar
31	Ningshu10	White	China	Modern cultivar
32	Xushu18	White	China	Modern cultivar
33	Ganshu9	White	China	Modern cultivar
34	Jiangnanshao	White	China	Local cultivar
35	Qingpizhong	White	China	Local cultivar
36	Suining16	White	China	Modern cultivar
37	Guangcaishu6	White	China	Modern cultivar
38	Xichengshu007	White	China	Modern cultivar
39	CA4	Yellow	China	Commercially available
40	3-15-53	White	China	Modern cultivar
41	2-16	White	China	Modern cultivar
42	Caishu5	White	China	Modern cultivar
43	P553	White	China	Modern cultivar
44	Ganshu22	White	China	Modern cultivar
45	SH5	White	China	Modern cultivar
46	CA12	White	China	Commercially available
47	CA7	White	China	Commercially available
48	Longshu599	White	China	Modern cultivar
49	CA6	Yellow	China	Commercially available
50	3-14-52	Yellow	China	Modern cultivar
51	Jishu26	Yellow	China	Modern cultivar
52	Jinguahuang	Yellow	China	Local cultivar
53	CA5	Yellow	China	Commercially available
54	Sanguishu15	White	China	Modern cultivar
55	Liushiri	White	China	Local cultivar
56	CA13	White	China	Commercially available
57	SH3	White	China	Modern cultivar
58	Xiangshu1	White	China	Modern cultivar
59	Jishu8088	White	China	Modern cultivar
60	2-190	White	China	Local cultivar
61	Xushu27	White	China	Modern cultivar
62	3-24	White	China	Local cultivar
63	Nanshu88	Yellow	China	Modern cultivar
64	Chuanshu383	White	China	Modern cultivar
65	CA11	Yellow	China	Commercially available
66	Chuanshu217	White	China	Modern cultivar
67	CA9	White	China	Commercially available
68	Chuanshu211	White	China	Modern cultivar
69	Baiguqilong	White	China	Local cultivar
70	Longshu14	White	China	Modern cultivar
71	Hainanzishu	Purple	China	Modern cultivar
72	CA3	Purple	China	Commercially available
73	Fushu404	Purple	China	Modern cultivar
74	Xuzishu9	Purple	China	Modern cultivar
75	Xuzishu8088	Purple	China	Modern cultivar
76	Rizi7	Purple	Japan	Introduced cultivar
77	Ribenzi	Purple	Japan	Introduced cultivar
78	Zisehongshao	Purple	China	Modern cultivar
79	Xuzishu201	Purple	China	Modern cultivar
80	Wanzishu56	Purple	China	Modern cultivar
81	Xuzishu7	Purple	China	Modern cultivar
82	Chuanzishu4	Purple	China	Modern cultivar
83	CA10	Purple	China	Commercially available
84	Chuanzishu3	Purple	China	Modern cultivar
85	Rizishu9	Purple	Japan	Introduced cultivar
86	1-24	Purple	China	Modern cultivar

**Table 2:** Nutrient composition of sweet potato root.

Nutrients	Range	Mean	SD	Coefficient of Variation (CV) %
Dry matter g/100 g	18.0–36.5	25.2	3.8	15.2
Protein g/100 g DW	2.15–7.11	3.55	1.00	28.3
Total starch g/100 g DW	22.6–69.7	48.5	8.3	17.1
Amylose ^1^ %	15.3–34.7	23.2	3.5	15.0
Resistant starch g/100 g DW	0.254–9.12	1.29	1.30	102
Fructose g/100 g DW	0.512–10.6	3.53	1.87	53.0
Glucose g/100 g DW	0.585–10.6	4.08	2.26	55.5
Sucrose g/100 g DW	2.75–36.2	14.7	4.9	33.2
TSS ^2^ g/100 g DW	10.3–40.0	22.3	4.8	21.3
TDF ^3^ g/100 g DW	7.99–26.0	16.9	2.9	17.0
Cyanidin mg/kg DW	0–708	61.3	150.1	245
Paeoniflorin mg/kg DW	0–1466	106	288	272
Pelargonidin mg/kg DW	0–23.3	1.24	3.58	288
Total anthocyanins mg/kg DW	0–2003	168	419	249
β-Carotene mg/kg DW	0–133	10.5	21.8	207
5-CQA ^4^ g/kg DW	0–0.850	0.182	0.143	78.4
3-CQA g/kg DW	0.0922–5.69	1.17	1.06	90.6
4-CQA g/kg DW	0–0.878	0.148	0.157	106
Caffeic acid g/kg DW	0.0220–0.678	0.177	0.100	56.4
3,4-diCQA g/kg DW	0.0536–2.02	0.569	0.423	74.5
3,5-diCQA g/kg DW	0.131–4.04	1.39	0.97	69.6
4,5-diCQA g/kg DW	0.0516–1.37	0.399	0.272	68.1
3,4,5-triCQA g/kg DW	0–0.979	0.120	0.135	112
Total CQAs g/kg DW	0.478–14.2	4.16	2.98	71.7
Potassium g/kg DW	5.56–24.8	12.4	4.2	33.6
Sodium g/kg DW	0.492–4.95	1.68	0.84	49.9
Calcium g/kg DW	0.638–6.33	2.56	0.95	37.2
Magnesium g/kg DW	0.373–2.16	0.981	0.284	29.0
Phosphorus g/kg DW	1.32–4.40	2.42	0.52	21.6
Iron g/kg DW	0.0731–0.593	0.186	0.081	43.8
Copper mg/kg DW	3.03–10.4	5.29	1.21	22.8
Zinc mg/kg DW	5.69–17.6	9.11	2.05	22.5
Manganese mg/kg DW	3.72–37.3	15.0	7.1	47.4
Selenium mg/kg DW	0.0294–0.247	0.0831	0.0399	48.2

^1^ Amylose % is on a % of total starch basis; ^2^ TSS: total soluble sugar; ^3^ TDF: total dietary fiber; ^4^ CQA: caffeoylquinic acid.

## Data Availability

The original contributions presented in the study are included in the article and Supplementary Materials, further inquiries can be directed to the corresponding author.

## Supplementary Materials

The following supporting information can be downloaded at: https://www.mdpi.com/article/10.3390/foods13223636/s1, Table S1: Nutrient composition in different sweet potato accessions; Table S2: Nutritional quality comparison of different flesh-colored sweet potato; Table S3: Nutritional quality comparison of sweet potato in six clusters; Table S4: Principal component analysis of 30 indicators; Table S5: Correlation analysis of thirty-four nutritional qualities. Supplementary Methods for Instrumental Parameters.
