# Supplementary material for: Comparative Analysis of Nutrients, Phytochemicals, and Minerals in Colored Sweet Potato (Ipomoea batatas L.) Roots
Source: Foods. 2024 Nov 14;13(22):3636. doi: 10.3390/foods13223636 (PMC11593716; doi:10.3390/foods13223636)
Supplement: Supplementary file 1 [file foods-13-03636-s001.zip › Supplementary methods for instrumental parameters.pdf]

Supplementary methods for instrumental parameters

S1. Soluble sugars

HPLC system (Agilent 1100, Agilent Technologies Inc., USA) with ZORBAX NH_2 column (250 mm×4.6 mm, 5 μm; Agilent, USA) and refractive index detector. The mobile phase was 85% (v/v) acetonitrile aqueous solution with a flow rate of 1 mL/min.

S2. β-Carotene quantification

HPLC system (Agilent 1100, Agilent Technologies Inc., USA) was equipped with Sunfire C18 column (250 mm × 4.6 mm, 5 μm; Waters, USA) and diode array detector (DAD). The mobile phase was ethanol, the flow rate was 1 mL/min and the absorbance was measured at 450 nm.

S3. Determination of polyphenol content

Chromatography was conducted on an Agilent 1290 system equipped with DAD detector (Agilent Technologies Inc., USA). HPLC separation was conducted on an ACQUITY UPLC HSS T3 column (2.1 mm × 100 mm, 1.8 μm; Waters, USA), and the column compartment was set at 30℃. An autosampler injection volume of 2 μL was used. The binary mobile phase was composed of water containing 0.1% (v/v) formic acid (A) and acetonitrile containing 0.1% (v/v) formic acid (B). The gradient elution was as follows: 10%−15% B from 0 to 2 min, 15%−20% B from 2 min to 4 min, 20%−22% B from 4 min to 4.5 min, 22%−35% B from 6 min to 7.2 min, 35%−60% B from 7.2 min to 8.5 min, 60% B from 8.5 min to 8.8 min, 60%−10% B from 8.8 min to 9.0 min, post-run for 2.5 min at a flow rate of 0.3 mL/min. The wavelength was set at 326 nm for monitoring CQAs.

S4. Acid hydrolysis of anthocyanins

Chromatography was conducted on an Agilent 1290 system, the configuration was the same as that in 2.6.4. The column used was ACQUITY BEH C18 (2.1 mm × 100 mm, 1.7 μm; Waters, USA). Mobile phase A was water (containing 1% formic acid), and B was acetonitrile (containing 1% formic acid). The gradient elution was as follows: 8%−21% B from 0 to 8 min, 21%−90% B from 8 min to 10 min, 90%−8% B from 10 min to 12 min, 8% B from 12 min to 16 min at a flow rate of 0.3 mL/min. The injection volume was 2.0 μL. The column temperature was 35℃. The detection wavelength was set at 530 nm.

S5. Mineral content

The optimized operation conditions for analysis of the diluted samples were as follows: radio frequency power 1500 W, plasma gas flow rate 18 L/min, auxiliary gas flow rate 1.2 L/min, atomizer flow rate 0.89 L/min, nebulization chamber temperature 2℃, Sampling cone: nickel.
