# Supplementary material for: Comparative Analysis of Nutrients, Phytochemicals, and Minerals in Colored Sweet Potato (Ipomoea batatas L.) Roots
Source: Foods. 2024 Nov 14;13(22):3636. doi: 10.3390/foods13223636 (PMC11593716; doi:10.3390/foods13223636)
Supplement: Supplementary file 1 [file foods-13-03636-s001.zip › Supplementary table.pdf]

Supplementary materials

Table S1 Nutrient composition in different sweetpotato accessions

Accession No.	Protein g/100g DW	Total starch g/100g DW	Amylose %	Resistant starch g/100g DW	TSS g/100g DW	TDF g/100g DW	Total anthocyanins mg/kg DW	β-Carotene mg/kg DW	Total CQAs g/kg DW	Potassium g/kg DW	Sodium g/kg DW	Calcium g/kg DW	Magnesium g/kg DW	Phosphorus g/kg DW	Iron g/kg DW	Copper mg/kg DW	Zinc mg/kg DW	Manganese mg/kg DW	Selenium mg/kg DW
1	2.94	42.41	24.34	0.55	28.68	17.70	0.00	23.54	2.87	12.60	2.40	2.52	0.91	1.85	0.18	4.01	7.98	24.90	0.03
2	2.84	52.17	20.58	0.41	24.70	11.42	0.00	0.34	1.86	12.72	1.72	1.08	0.71	2.80	0.12	4.86	9.47	6.75	0.07
3	2.61	42.69	17.26	0.44	28.51	14.45	0.00	26.08	1.84	6.51	1.82	2.85	1.14	2.05	0.10	3.71	7.33	28.35	0.06
4	2.66	53.29	17.92	0.43	21.51	13.69	0.00	8.85	2.50	9.35	1.98	2.19	0.89	2.33	0.14	5.29	6.82	16.64	0.05
5	2.45	39.68	19.92	0.37	28.54	16.30	0.00	39.35	4.01	12.71	1.57	3.39	0.94	2.28	0.58	4.06	8.02	18.27	0.11
6	4.04	41.87	21.39	0.71	26.12	20.85	0.00	40.85	1.83	6.38	2.96	2.28	0.95	1.55	0.18	5.47	7.34	18.31	0.05
7	4.17	39.34	17.10	1.32	22.64	18.15	0.00	20.50	3.68	17.34	1.49	1.96	1.00	2.80	0.17	5.35	9.55	10.26	0.07
8	3.10	43.30	25.88	1.33	28.66	17.54	0.00	129.94	3.08	12.50	1.32	2.87	1.18	3.00	0.18	4.82	7.84	13.97	0.08
9	2.48	57.15	22.65	1.50	17.42	15.87	0.00	17.49	2.79	9.02	2.26	2.29	0.87	2.17	0.32	3.25	6.88	18.22	0.09
10	2.15	51.28	27.47	1.48	21.89	16.06	0.00	51.80	3.00	6.84	3.26	2.17	0.73	2.12	0.24	5.62	8.77	14.93	0.06
11	2.67	42.19	25.17	1.44	26.50	19.34	0.00	63.61	1.12	9.76	0.89	2.39	0.68	1.34	0.14	4.23	6.53	16.54	0.06
12	4.10	50.30	23.09	0.58	18.95	19.05	0.00	15.44	3.72	9.35	2.87	2.00	0.70	2.43	0.30	5.48	8.97	14.11	0.07
13	3.41	34.54	19.26	0.33	28.79	21.54	0.00	114.73	7.55	8.78	2.20	3.49	0.89	2.61	0.23	5.66	7.71	11.70	0.06
14	3.53	49.38	24.23	0.48	20.66	17.38	0.00	63.01	3.50	9.34	2.59	2.89	0.98	2.91	0.25	5.37	10.09	12.70	0.09
15	7.01	22.76	25.72	0.75	25.60	25.48	0.00	7.23	6.71	24.79	3.15	3.83	1.40	4.38	0.24	10.44	17.55	13.25	0.14
16	2.57	58.18	27.45	2.01	16.35	17.08	0.00	0.98	2.18	10.01	1.16	1.17	0.48	2.19	0.15	4.89	5.95	8.53	0.07
17	3.50	37.01	23.07	0.37	29.76	19.28	0.00	0.31	5.91	12.55	0.87	2.25	0.86	2.64	0.33	4.17	10.85	16.73	0.09
18	2.80	42.05	23.06	0.72	23.50	18.18	0.00	4.06	4.56	10.21	2.53	3.12	0.91	2.49	0.30	4.97	6.30	18.93	0.10
19	3.72	42.58	21.77	0.69	25.95	17.74	0.00	5.82	4.03	15.26	1.92	2.11	0.86	1.41	0.15	4.76	6.55	12.02	0.04
20	4.78	36.75	21.66	0.93	21.85	22.00	0.00	7.07	7.26	22.63	1.63	3.93	1.10	3.29	0.31	8.86	12.31	16.89	0.23
21	3.11	47.71	22.48	1.10	22.40	17.53	0.00	9.14	3.09	11.84	1.59	2.43	0.82	2.24	0.22	4.36	7.64	14.16	0.07
22	3.99	40.09	23.48	0.55	25.71	21.32	0.00	10.85	3.39	13.50	1.08	2.83	0.84	2.48	0.23	7.04	9.65	17.47	0.10
23	4.12	22.63	20.83	0.26	40.04	19.83	0.00	11.80	10.41	21.21	2.03	4.60	1.49	2.59	0.26	7.48	9.73	32.84	0.09
24	2.70	47.25	15.60	0.57	20.75	18.38	0.00	13.34	1.47	15.20	1.51	2.84	0.86	2.33	0.30	4.22	10.98	12.24	0.11
25	2.90	45.54	20.41	0.35	23.49	17.33	0.00	14.11	2.82	17.20	0.73	2.70	0.88	2.40	0.19	4.71	10.55	12.52	0.04
26	4.00	49.09	24.00	1.03	19.29	16.33	0.00	17.45	4.45	16.70	1.07	1.66	0.89	2.61	0.26	6.78	10.48	13.89	0.10

27	3.20	45.53	23.04	0.53	22.62	17.70	0.00	19.08	6.96	11.33	1.68	2.99	1.53	2.47	0.19	4.56	8.14	34.26	0.09
28	3.14	37.07	30.71	0.36	29.96	20.14	0.00	19.89	5.65	10.52	2.86	2.52	1.01	3.03	0.28	5.13	10.03	10.75	0.13
29	3.58	38.86	23.23	0.62	26.69	17.38	0.00	21.57	4.47	15.60	1.20	3.57	1.14	2.34	0.17	4.81	8.37	23.49	0.07
30	2.93	51.62	18.06	0.62	24.08	15.72	0.00	22.05	0.49	6.65	1.37	3.12	0.89	2.28	0.22	4.51	9.91	9.72	0.09
31	2.90	52.77	24.27	0.71	22.25	14.47	0.00	0.00	1.56	11.72	1.00	1.98	1.06	2.30	0.12	3.03	7.47	13.02	0.05
32	3.38	56.41	33.50	2.18	13.32	12.64	0.00	0.00	1.82	9.50	1.34	1.76	0.81	2.13	0.12	5.35	7.06	14.51	0.09
33	2.79	40.84	26.51	0.65	22.00	20.68	0.00	0.25	5.81	20.57	0.66	3.25	0.79	2.51	0.23	4.14	10.31	9.71	0.10
34	2.82	54.02	22.20	0.69	18.90	16.85	0.00	0.25	2.46	11.64	0.81	2.37	0.95	2.16	0.14	4.34	9.71	18.18	0.05
35	2.62	47.52	23.27	0.53	30.35	14.07	0.00	0.39	2.53	11.62	3.14	1.38	0.60	2.18	0.17	4.46	8.44	8.60	0.05
36	4.03	57.78	23.25	1.40	19.05	12.99	0.00	0.21	1.31	9.95	1.58	1.53	0.81	2.68	0.12	6.30	9.22	6.13	0.05
37	3.95	41.13	23.83	0.60	24.74	18.72	0.00	3.42	8.42	12.52	1.15	2.33	0.95	2.71	0.35	6.20	8.87	17.04	0.07
38	2.52	57.40	23.94	0.67	24.08	12.83	0.00	1.21	2.56	10.75	2.95	1.74	0.58	2.28	0.10	4.23	6.04	5.23	0.08
39	2.63	59.06	23.53	0.66	21.44	13.27	0.00	17.55	4.14	9.86	1.17	2.81	0.89	1.86	0.15	5.20	7.40	18.18	0.04
40	3.48	39.08	23.20	0.27	28.99	18.18	0.00	4.92	5.42	21.26	0.59	2.71	1.38	3.07	0.17	4.60	10.32	20.69	0.13
41	3.97	42.04	19.80	0.51	25.53	18.72	0.00	0.40	4.49	12.56	1.71	4.47	1.10	1.85	0.23	3.92	8.93	20.13	0.06
42	3.34	39.76	19.57	0.32	28.26	20.17	0.00	0.81	5.65	13.58	2.52	4.38	1.55	2.42	0.18	6.31	8.49	37.27	0.07
43	3.87	45.55	20.79	4.14	21.99	21.38	0.00	0.00	3.94	15.19	1.14	3.47	0.97	2.70	0.33	6.00	15.27	16.41	0.15
44	3.44	51.39	24.34	0.55	20.64	17.43	0.00	0.00	2.43	12.24	1.12	2.53	0.68	2.18	0.16	5.21	8.36	10.51	0.07
45	5.06	43.92	20.97	0.95	20.16	20.07	0.00	0.00	3.24	16.68	0.55	6.32	1.50	3.21	0.26	7.95	14.07	28.74	0.18
46	3.76	54.64	23.08	0.84	22.11	13.03	0.00	0.00	2.04	13.69	1.89	1.47	0.77	2.57	0.16	6.74	9.22	8.18	0.05
47	3.02	55.37	28.75	0.57	17.06	15.05	0.00	0.38	1.27	16.77	0.66	3.00	0.92	2.30	0.13	5.34	7.78	9.14	0.08
48	2.62	53.99	27.58	0.89	24.32	14.86	0.00	0.51	1.82	6.54	1.59	2.70	0.83	1.87	0.15	4.33	5.78	14.86	0.05
49	3.44	56.75	29.44	0.55	19.23	12.16	0.00	7.31	1.83	8.49	0.74	0.67	0.43	1.87	0.10	4.77	7.00	3.74	0.07
50	2.95	46.10	22.54	0.69	25.42	18.04	0.00	19.60	5.08	5.56	2.35	3.03	0.98	2.31	0.15	6.49	9.37	10.43	0.06
51	2.47	46.77	19.79	0.47	25.30	16.16	0.00	9.25	1.73	14.55	1.39	2.15	0.98	2.61	0.16	5.27	10.79	8.31	0.09
52	6.40	53.85	20.63	0.59	17.76	14.86	0.00	2.39	1.81	7.80	1.98	3.57	1.55	1.46	0.13	6.24	6.69	36.69	0.05
53	3.08	52.16	27.16	0.83	23.39	16.43	0.00	12.36	4.16	14.07	1.10	2.83	1.00	2.11	0.14	5.55	7.96	21.45	0.08
54	7.08	44.85	26.43	0.81	16.92	21.31	0.00	0.41	0.88	7.64	4.93	5.57	2.11	2.34	0.22	6.76	8.85	21.86	0.05
55	4.17	57.01	30.25	0.93	19.79	15.54	0.00	0.39	1.42	10.72	1.19	1.72	0.61	1.67	0.13	6.01	6.15	6.85	0.06
56	4.03	43.20	28.13	0.59	24.70	19.93	0.00	1.15	2.89	11.23	1.94	2.89	1.05	2.84	0.39	5.22	11.18	14.66	0.09
57	3.68	43.91	23.73	1.42	26.02	18.13	0.00	0.28	1.97	10.75	0.53	2.97	1.24	2.29	0.20	4.58	8.85	7.48	0.21
58	2.25	57.26	24.62	1.43	17.64	17.61	0.00	0.30	0.96	7.40	2.55	3.41	1.25	2.63	0.20	4.68	7.35	14.90	0.06

59	2.97	63.56	23.94	2.22	16.43	12.67	0.00	1.65	1.49	7.60	1.02	1.39	0.56	1.75	0.11	4.69	9.77	18.73	0.06
60	2.25	69.66	26.53	1.52	10.31	11.44	0.00	0.00	0.74	10.87	0.50	1.78	0.79	2.01	0.13	3.47	7.62	11.59	0.07
61	3.46	51.43	25.65	1.36	18.54	19.29	0.00	0.59	1.68	11.41	2.10	2.02	0.92	2.36	0.17	5.44	10.13	11.33	0.07
62	2.91	50.00	25.62	1.01	23.53	18.17	0.00	0.44	3.72	9.80	1.28	2.70	0.91	2.19	0.16	4.60	7.57	24.10	0.08
63	3.59	51.68	27.12	0.78	20.41	18.59	0.00	6.52	5.39	8.58	2.02	3.18	0.96	1.98	0.26	5.83	7.35	33.49	0.07
64	2.79	57.53	27.51	2.27	15.05	18.86	0.00	0.00	1.73	12.78	1.42	2.15	0.77	2.59	0.22	6.08	9.41	11.50	0.07
65	3.37	51.83	26.54	0.57	22.93	15.65	0.00	13.80	2.80	11.12	1.62	2.46	0.95	2.27	0.14	5.79	9.19	17.35	0.08
66	5.28	57.23	26.11	1.50	14.96	15.11	0.00	0.00	1.61	11.61	1.29	1.95	0.77	2.43	0.14	7.05	9.51	10.90	0.06
67	3.18	67.97	26.58	1.63	13.61	8.25	0.00	0.00	3.17	8.07	1.37	0.92	0.74	1.91	0.10	4.69	6.61	9.36	0.04
68	2.90	55.29	20.00	0.84	15.97	20.32	0.00	0.00	1.81	17.19	1.02	2.96	0.83	1.96	0.10	4.65	11.59	8.52	0.07
69	3.01	61.21	26.62	1.75	19.35	13.87	0.00	0.00	1.03	7.84	2.15	1.98	0.80	2.07	0.10	5.58	7.04	20.03	0.04
70	3.11	54.62	20.49	3.01	19.53	14.88	0.00	0.00	0.73	12.78	0.62	2.64	0.96	2.08	0.14	3.88	8.60	17.30	0.06
71	4.10	57.38	22.19	1.40	17.04	13.05	456.74	0.00	5.82	13.78	0.59	1.61	0.90	2.70	0.09	4.86	7.48	9.52	0.08
72	2.34	57.14	25.80	8.73	17.03	15.21	330.64	0.00	5.66	6.83	1.10	1.91	0.74	1.61	0.13	3.68	9.29	13.22	0.06
73	3.07	39.34	28.40	2.52	28.45	17.83	819.21	0.00	11.07	16.09	0.55	2.63	1.07	2.86	0.15	5.43	12.31	9.24	0.11
74	4.38	50.75	22.50	3.12	17.62	16.64	1782.30	0.00	11.67	13.58	1.96	3.35	1.21	2.40	0.14	6.03	11.72	12.06	0.07
75	3.04	56.18	22.85	2.77	17.35	13.30	1503.47	0.00	14.12	10.39	1.32	1.52	0.98	2.01	0.10	4.65	8.12	8.83	0.07
76	4.71	48.09	19.80	1.28	22.35	18.22	710.27	0.00	8.45	16.38	1.72	2.57	1.27	3.50	0.11	5.97	9.97	16.44	0.06
77	5.62	43.67	17.99	0.78	23.14	16.78	456.21	0.00	5.69	15.41	1.46	1.75	1.32	3.70	0.17	6.76	11.47	10.72	0.14
78	4.00	49.69	19.01	2.47	22.25	13.78	329.12	0.00	3.35	13.07	2.22	1.62	0.78	2.75	0.16	5.16	11.16	11.11	0.15
79	3.52	43.81	21.52	3.59	24.04	16.43	1548.88	0.00	12.42	11.84	3.27	1.89	1.30	2.94	0.17	4.89	10.99	9.30	0.13
80	3.40	51.73	15.80	5.38	21.02	15.59	668.88	0.00	6.14	8.01	2.57	1.59	0.90	2.05	0.07	4.70	10.02	6.64	0.06
81	3.66	39.14	21.07	1.72	29.04	16.61	373.07	0.00	3.03	14.65	2.48	1.26	0.88	2.71	0.24	4.17	9.78	8.49	0.12
82	4.67	46.31	20.43	0.79	22.64	18.48	615.67	0.00	5.86	15.23	1.89	2.36	1.21	2.97	0.17	6.47	9.73	11.84	0.11
83	6.31	46.90	20.02	1.07	21.74	17.66	661.25	0.00	7.78	15.27	1.61	3.65	1.85	3.47	0.09	7.36	12.08	20.02	0.09
84	3.76	51.94	21.82	5.63	20.38	17.54	996.53	0.00	8.27	6.53	3.86	2.31	1.19	2.08	0.13	4.38	10.56	17.28	0.05
85	3.83	57.09	19.28	1.83	14.93	15.51	1952.99	0.00	10.67	20.64	0.91	2.01	0.86	2.80	0.11	4.58	9.14	6.55	0.09
86	4.85	42.15	20.64	0.78	23.96	19.09	1254.49	0.00	10.63	22.85	0.66	2.58	1.09	3.43	0.12	6.12	10.25	10.46	0.25

Note:Accession No. refers to Table 1; All the data listed in the table are average values.

Table S2 Nutritional quality comparison of different flesh-colored sweetpotato

Nutrients	WFSP	YFSP	OFSP	PFSP
Dry matter g/100g	25.8^{a}±3.8	24.3^{b}±4.3	23.7^{b}±3.2	25.7^{a}±3.1
Protein g/100g DW	3.51^{b}±1.07	3.45^{b}±0.86	2.99^{c}±0.57	4.08^{a}±0.98
Total starch g/100g DW	50.4^{a}±9.1	47.2^{b}±8.1	43.0^{c}±4.7	48.8^{ab}±6.1
Amylose %	24.3^{a}±3.2	22.9^{b}±3.8	22.8^{b}±3.3	21.2^{c}±2.9
Resistant starch g/100g DW	1.13^{b}±0.79	0.692^{c}±0.306	0.791^{bc}±0.464	2.74^{a}±2.15
TSS g/100g DW	21.1^{c}±4.7	23.3^{b}±4.9	26.5^{a}±3.0	21.4^{c}±3.9
TDF g/100g DW	16.8^{b}±3.5	17.1^{ab}±2.2	17.9^{a}±2.3	16.4^{b}±1.8
Total anthocyanins mg/kg DW	0^{b}±0	0^{b}±0	0^{b}±0	904^{a}±528
β-Carotene mg/kg DW	1.32^{c}±2.28	14.4^{b}±5.4	61.4^{a}±36.0	0^{c}±0
Total CQAs g/kg DW	2.94^{c}±1.93	3.83^{b}±2.14	3.20^{bc}±1.79	8.17^{a}±3.23
Potassium g/kg DW	12.5^{b}±4.2	12.2^{b}±4.0	9.49^{c}±2.53	13.8^{a}±4.4
Sodium g/kg DW	1.58^{b}±0.90	1.63^{b}±0.63	2.11^{a}±0.75	1.76^{ab}±0.93
Calcium g/kg DW	2.60^{a}±1.16	2.68^{a}±0.78	2.76^{a}±0.45	2.16^{b}±0.75
Magnesium g/kg DW	0.944^{b}±0.312	0.983^{b}±0.261	0.933^{b}±0.156	1.10^{a}±0.27
Phosphorus g/kg DW	2.39^{b}±0.51	2.32^{bc}±0.34	2.19^{c}±0.55	2.75^{a}±0.59
Iron g/kg DW	0.184^{b}±0.076	0.207^{ab}±0.063	0.231^{a}±0.134	0.134^{c}±0.041
Copper mg/kg DW	5.35^{a}±1.42	5.37^{a}±1.01	4.77^{b}±0.75	5.32^{ab}±1.01
Zinc mg/kg DW	9.03^{b}±2.51	8.89^{b}±1.36	7.96^{c}±0.97	10.3^{a}±1.3
Manganese mg/kg DW	14.4^{b}±6.5	17.8^{a}±9.2	17.7^{a}±5.4	11.4^{c}±3.7
Selenium mg/kg DW	0.0814^{b}±0.0441	0.0794^{b}±0.0223	0.0663^{b}±0.0226	0.102^{a}±0.049

Note: Values followed by the same lowercase letters are not significantly different (P<0.05) based on ANOVA test.

Table S3 Nutritional quality comparison of sweetpotato in six cluster

Nutrients	I	II	III	IV	V	VI
Dry matter g/100g	26.3^{b}±2.8	25.9^{b}±3.8	22.7^{cd}±2.8	29.4^{a}±2.8	22.1^{cd}±2.8	24.4^{bc}±3.4
Protein g/100g DW	4.11^{a}±1.07	2.91^{c}±0.58	3.41^{bc}±0.43	3.39^{bc}±0.80	3.21^{c}±0.64	4.03^{ab}±1.47
Total starch g/100g DW	49.5^{b}±6.1	48.3^{b}±6.3	42.2^{c}±7.2	58.9^{a}±5.3	46.4^{bc}±5.0	47.7^{b}±9.0
Amylose %	21.4^{c}±3.1	22.8^{bc}±2.8	24.0^{b}±3.2	26.8^{a}±3.0	19.0^{c}±1.9	23.6^{b}±2.6
Resistant starch g/100g DW	2.83^{a}±2.34	0.817^{b}±0.422	0.580^{b}±0.198	1.38^{b}±0.58	0.990^{b}±0.772	1.24^{b}±0.98
TSS g/100g DW	20.8^{bc}±3.7	24.4^{a}±4.1	26.0^{a}±4.4	17.7^{d}±3.9	24.0^{ab}±2.5	20.5^{c}±3.9
TDF g/100g DW	16.5^{b}±1.8	16.4^{b}±2.4	18.7^{a}±1.5	13.6^{c}±2.5	15.9^{b}±2.3	18.7^{a}±3.0
Total anthocyanins mg/kg DW	983^{a}±531	0^{b}±0	0^{b}±0	0^{b}±0	87.8^{b}±162.9	0^{b}±0
β-Carotene mg/kg DW	0^{c}±0	33.9^{a}±33.6	14.2^{b}±26.3	0.850^{bc}±2.004	9.95^{bc}±9.08	2.90^{bc}±4.88
Total CQAs g/kg DW	8.88^{a}±2.87	2.84^{c}±0.83	5.44^{b}±1.93	1.76^{c}±0.63	2.30^{c}±1.09	2.85^{c}±1.98
Potassium g/kg DW	13.8^{a}±4.8	9.90^{b}±2.14	12.5^{ab}±3.9	9.78^{b}±2.05	13.9^{a}±3.4	14.4^{a}±5.0
Sodium g/kg DW	1.68^{ab}±0.99	2.19^{a}±0.77	1.76^{ab}±0.62	1.30^{bc}±0.44	1.61^{ab}±0.54	1.49^{bc}±1.13
Calcium g/kg DW	2.27^{bc}±0.65	2.41^{b}±0.51	3.12^{a}±0.75	1.66^{c}±0.57	2.09^{bc}±0.75	3.19^{a}±1.20
Magnesium g/kg DW	1.14^{a}±0.27	0.857^{bc}±0.177	1.05^{ac}±0.23	0.698^{c}±0.136	0.872^{bc}±0.095	1.15^{a}±0.34
Phosphorus g/kg DW	2.75^{a}±0.65	2.17^{cd}±0.44	2.37^{bc}±0.38	2.10^{d}±0.31	2.59^{abc}±0.21	2.57^{ab}±0.63
Iron g/kg DW	0.125^{b}±0.032	0.217^{a}±0.120	0.230^{a}±0.078	0.129^{b}±0.024	0.195^{a}±0.057	0.198^{a}±0.067
Copper mg/kg DW	5.42^{ab}±1.05	4.64^{b}±0.74	5.31^{ab}±0.94	5.31^{ab}±1.01	4.78^{b}±0.46	5.93^{a}±1.85
Zinc mg/kg DW	10.2^{a}±1.4	7.74^{b}±1.04	8.72^{b}±1.38	7.64^{b}±1.42	10.3^{a}±0.7	10.3^{a}±2.9
Manganese mg/kg DW	11.6^{b}±4.0	16.2^{ab}±5.6	20.4^{a}±8.9	11.1^{b}±4.8	9.93^{b}±2.01	16.5^{a}±7.2
Selenium mg/kg DW	0.0971^{a}±0.0508	0.0649^{b}±0.0207	0.0793^{ab}±0.0208	0.0600^{b}±0.0139	0.0930^{ab}±0.0340	0.103^{a}±0.057

Note: Values followed by the same lowercase letters are not significantly different ($P<0.05$) based on ANOVA test.

Table S4 Principal component analysis of 30 indicators

Index	1	2	3	4	5	6	7
Protein	0.459	0.366	-0.182	0.350	0.487	0.0953	0.0114
Total starch	-0.497	-0.702	-0.257	0.106	0.239	-0.0213	0.116
Resistant starch	0.169	-0.538	0.317	0.251	0.169	0.0629	0.253
amylose	-0.273	-0.0982	-0.282	-0.134	0.00427	0.477	0.515
Fructose	-0.142	0.243	0.840	0.210	-0.202	-0.123	-0.0270
Glucose	-0.263	0.234	0.849	0.189	-0.0715	-0.0486	0.0381
Sucrose	0.406	0.287	-0.428	-0.485	-0.222	0.0473	-0.319
TDF	0.397	0.701	0.212	0.0667	-0.0365	0.101	0.125
β-Carotene	-0.0821	0.250	0.235	-0.383	-0.215	0.308	-0.399
Cyanidin	0.648	-0.512	0.277	0.246	0.168	-0.0239	-0.126
Paeoniflorin	0.710	-0.575	0.0544	0.0817	0.0514	-0.0493	-0.113
Pelargonidin	0.688	-0.545	0.133	0.128	0.134	-0.0364	-0.0480
5-CQA	0.850	-0.126	0.0960	-0.306	-0.0507	-0.0134	-0.0184
3-CQA	0.896	-0.291	0.0289	-0.110	-0.0671	-0.0637	-0.0623
4-CQA	0.867	-0.390	0.0481	-0.0376	-0.0224	-0.0543	-0.0389
Caffeic acid	0.476	0.0915	0.0709	-0.337	-0.122	-0.186	0.479
3,4-diCQA	0.790	-0.0523	0.266	-0.240	-0.106	0.195	0.128
3,5-diCQA	0.887	-0.161	0.151	-0.224	-0.0863	0.0652	0.134
4,5-diCQA	0.893	-0.0764	0.0985	-0.202	-0.0974	0.0917	0.0667
3,4,5-triCQA	0.724	-0.194	-0.158	-0.0698	0.0554	0.100	0.0553
Potassium	0.565	0.302	-0.377	0.252	-0.360	-0.244	-0.0218
Sodium	0.0713	0.163	0.391	-0.0745	0.472	0.559	-0.227
Calcium	0.301	0.681	0.0749	-0.126	0.345	-0.253	0.106
Magnesium	0.499	0.451	0.00992	-0.00883	0.564	-0.220	-0.151
Phosphorus	0.594	0.357	-0.261	0.391	-0.162	0.167	-0.190
Iron	0.0817	0.586	0.231	-0.0744	-0.305	0.162	0.232
Copper	0.436	0.467	-0.351	0.237	0.264	0.329	0.121
Zinc	0.546	0.336	0.00716	0.590	-0.0756	0.00606	0.0932
Manganese	0.0772	0.460	0.0188	-0.504	0.466	-0.349	0.111
Selenium	0.470	0.307	-0.195	0.403	-0.311	-0.0721	0.0217
Characteristic value	9.35	4.79	2.82	2.20	1.87	1.25	1.14
Variance contribution rate %	31.2	16.0	9.39	7.34	6.22	4.16	3.81
Cumulative variance contribution rate %	31.2	47.1	56.5	63.9	70.1	74.2	78.0

Table S5 Correlation analysis of thirty-four nutritional qualities

Correlation	Dry matter	Protein	Total starch	Resistant starch	Amylose	Fructose	Glucose	Sucrose	TSS	TDF	β-Carotene	Cyanidin	Paeoniflorin	Pelargonidin	Total anthocyanins
Protein	-0.168**														
Total starch	0.754**	-0.330**													
Resistant starch	0.176	-0.091	0.282**												
Amylose	0.312**	-0.126	0.250*	0.007											
Fructose	-0.607**	-0.101	-0.383**	0.031	-0.298**										
Glucose	-0.526**	-0.125	-0.275*	0.061	-0.119	0.900**									
Sucrose	-0.141	0.065	-0.513**	-0.364**	-0.056	-0.363**	-0.487**								
TSS	-0.633**	-0.032	-0.807**	-0.331**	-.231*	0.450**	0.331**	0.650**							
TDF	-0.596**	0.368**	-0.747**	-0.155	-0.100	0.225*	0.228*	0.191	0.392**						
β-Carotene	-0.192	-0.166	-0.274**	-0.179	-0.034	0.147	0.194	0.174	0.328**	0.193					
Cyanidin	0.038	0.225*	0.017	0.512**	-0.274*	0.050	-0.051	-0.090	-0.097	-0.035	-0.198				
Paeoniflorin	0.188	0.161	0.058	0.349**	-0.184	-0.187	-0.240*	0.032	-0.155	-0.087	-0.178	0.778**			
Pelargonidin	0.113	0.171	0.042	0.428**	-0.164	-0.128	-0.173	-0.013	-0.145	-0.040	-0.168	0.886**	0.869**		
Total anthocyanins	0.143	0.192	0.046	0.427**	-0.226*	-0.112	-0.185	-0.010	-0.143	-0.073	-0.195	0.901**	0.974**	0.923**	
5-CQA	-0.162	0.206	-0.422**	0.185	-0.212	-0.104	-0.226*	0.449**	0.312**	0.239*	0.041	0.568**	0.617**	0.625**	0.633**
3-CQA	-0.035	0.218*	-0.288**	0.259*	-0.206	-0.178	-0.256*	0.350**	0.167	0.144	-0.085	0.657**	0.837**	0.716**	0.817**
4-CQA	0.034	0.198	-0.190	0.344**	-0.196	-0.182	-0.246*	0.243**	0.060	0.072	-0.136	0.705**	0.889**	0.776**	0.871**
Caffeic acid	-0.188	0.149	-0.330**	-0.044	-0.072	0.046	-0.086	0.187	0.168	0.199	-0.043	0.140	0.182	0.203	0.177
3,4-diCQA	-0.239*	0.175	-0.437**	0.217*	-0.154	0.029	-0.046	0.301**	0.298**	0.315**	0.053	0.501**	0.514**	0.468**	0.537**
3,5-diCQA	-0.139	0.226*	-0.382**	0.213*	-0.140	-0.056	-0.171	0.336**	0.241*	0.272*	-0.049	0.614**	0.673**	0.624**	0.688**
4,5-diCQA	-0.174	0.271*	-0.431**	0.179	-0.208	-0.112	-0.213*	0.376**	0.240*	0.319**	-0.009	0.569**	0.629**	0.578**	0.641**
3,4,5-triCQA	0.002	0.312**	-0.166	0.137	-0.155	-0.308**	-0.388**	0.260*	-0.040	0.135	-0.048	0.468**	0.618**	0.658**	0.598**
Total CQAs	-0.120	0.240*	-0.377**	0.240*	-0.189	-0.114	-0.217*	0.363**	0.224*	0.241*	-0.048	0.646**	0.756**	0.684**	0.757**
Potassium	-0.261*	0.346**	-0.487**	-0.214*	-0.201	-0.164	-0.297**	0.416**	0.221*	0.396**	-0.165	0.142	0.215*	0.175	0.200
Sodium	-0.257*	0.208	-0.192	0.052	-0.054	0.187	0.213*	-0.013	0.162	0.197	0.110	0.153	-0.020	0.033	0.042
Calcium	-0.388**	0.369**	-0.507**	-0.226*	-0.155	0.053	0.104	0.188	0.263*	0.629**	0.123	-0.113	-0.121	-0.044	-0.124
Magnesium	-0.316**	0.607**	-0.449**	-0.092	-0.245*	-0.054	-0.025	0.243*	0.216*	0.423**	-0.027	0.198	0.165	0.163	0.186
Phosphorus	-0.231*	0.475**	-0.459**	-0.154	-0.176**	-0.104	-0.221*	0.340**	0.202	0.381**	-0.014	0.224*	0.224*	0.173	0.236*
Iron	-0.416**	0.011	-0.477**	-0.225*	-0.034	0.263*	0.238*	0.103	0.322**	0.483**	0.199	-0.273*	-0.260*	-0.240*	-0.279**

Copper	-0.229*	0.716**	-0.358**	-0.167	0.028	-0.221*	-0.253*	0.252*	0.051	0.429**	-0.056	0.005	0.005	0.026	0.006
Zinc	-0.329**	0.450**	-0.433**	0.166	-0.249*	0.109	0.030	0.060	0.118	0.493**	-0.162	0.272*	0.183	0.251*	0.225*
Manganese	-0.163	0.168	-0.273*	-0.188	-0.098	-0.051	-0.023	0.265*	0.240*	0.270*	0.063	-0.205	-0.234*	-0.189	-0.236*
Selenium	-0.243*	0.295**	-0.401**	-0.022	-0.119	-0.032	-0.148	0.2546*	0.168	0.372**	-0.092	0.175	0.187	0.161	0.193

Note: *correlation is significant at $P<0.05$, **correlation is highly significant at $P<0.01$.

Continued table

Correlation	5-CQA	3-CQA	4-CQA	Caffeic acid	3,4-diCQA	3,5-diCQA	4,5-diCQA	3,4,5-triCQA	Total CQAs	Potassium	Sodium	Calcium	Magnesium	Phosphorus	Iron	Copper	Zinc	Manganese
3-CQA	0.822**																	
4-CQA	0.777**	0.979**																
Caffeic acid	0.483**	0.371**	0.338**															
3,4-diCQA	0.745**	0.742**	0.723**	0.452**														
3,5-diCQA	0.841**	0.877**	0.828**	0.551**	0.856**													
4,5-diCQA	0.838**	0.815**	0.778**	0.495**	0.877**	0.903**												
3,4,5-triCQA	0.600**	0.643**	0.660**	0.395**	0.484**	0.637**	0.593**											
Total CQAs	0.879**	0.952**	0.921**	0.512**	0.874**	0.971**	0.923**	0.680**										
Potassium	0.369**	0.406**	0.359**	0.257*	0.259*	0.322**	0.442**	0.306**	0.385**									
Sodium	0.063	-0.035	-0.024	-0.098	0.203	0.069	0.107	0.002	0.047	-0.329**								
Calcium	0.214*	0.067	0.018	0.223*	0.165	0.122	0.180	0.108	0.127	0.240*	0.129							
Magnesium	0.319**	0.350**	0.303**	0.166	0.248*	0.311**	0.308**	0.220*	0.335**	0.241*	0.269	0.702**						
Phosphorus	0.309**	0.396**	0.346**	0.136	0.287**	0.388**	0.438**	0.337**	0.400**	0.614**	0.041	0.212	0.423**					
Iron	0.015	-0.087	-0.156	0.147	0.170	0.075	0.112	0.024	0.026	0.124	0.144	0.365**	0.078	0.215*				
Copper	0.219*	0.158	0.136	0.174	0.253*	0.217*	0.286**	0.347**	0.228*	0.400**	0.169	0.378**	0.394**	0.554**	0.112			
Zinc	0.261*	0.319**	0.323**	0.129	0.315**	0.302**	0.347**	0.291**	0.335**	0.559**	0.001	0.315**	0.344**	0.669**	0.233*	0.520**		
Manganese	0.126	-0.015	-0.085	0.208	0.074	0.067	0.080	-0.032	0.042	-0.051	0.121	0.599**	0.541**	-0.123	0.201	0.141	-0.076	
Selenium	0.206	0.325**	0.285**	0.088	0.248*	0.266*	0.283**	0.214*	0.300**	0.541**	-0.187	0.255*	0.252*	0.597**	0.302**	0.339**	0.531**	-0.075
